# Pregnancy, delivery, and postpartum period in infantile liver failure syndrome type 2 due to variants in *NBAS*

**DOI:** 10.1002/jmd2.12362

**Published:** 2023-02-15

**Authors:** Bianca Peters, Felix Wiemers, Dominic Lenz, Stefan Kölker, Georg F. Hoffmann, Siegmund Köhler, Christian Staufner

**Affiliations:** ^1^ Division of Pediatric Neurology and Metabolic Medicine, Center for Child and Adolescent Medicine University of Heidelberg Heidelberg Germany; ^2^ Center of Obstetrics and Gynecology University of Marburg Marburg Germany

**Keywords:** acute liver failure, birth, infantile liver failure syndrome type 2, neuroblastoma amplified sequence, pregnancy

## Abstract

Biallelic pathogenic variants in the neuroblastoma amplified sequence (*NBAS*) gene affecting the Sec39 domain are associated with a predominant hepatic phenotype named infantile liver failure syndrome type 2 (ILFS2). Individuals are at risk of developing life‐threatening acute liver failure episodes, most likely triggered by febrile infections. Pregnancy, delivery, and the postpartum period are well known triggers of decompensation in different inherited metabolic diseases and therefore entail a potential risk also for individuals with ILFS2. We studied pregnancy, birth, and postpartum period in a woman with ILFS2 (homozygous for the *NBAS* variant c.2708 T > G, p.(Leu903Arg)). During two pregnancies there were no complications associated with the underlying genetic condition. Two healthy boys were born by cesarean section. To reduce the risk of fever and febrile infections, we avoided prolonged labor, epidural analgesia, and breastfeeding. Maternal body temperature and liver function were closely monitored. In case of elevated body temperature, antipyretic treatment (acetaminophen, metamizole) was given without delay. Alanine and aspartate aminotransferases as well as liver function remained normal throughout the observation period. Hence, pregnancy and childbirth are feasible in women with ILFS2 under careful monitoring.

**Synopsis**

Avoidance of potential risk factors, careful medical assistance and monitoring by an interdisciplinary team, enables safe pregnancy and childbirth in women with infantile liver failure syndrome type 2 (ILFS2) due to variants in *NBAS*.

## 1 INTRODUCTION

Experience with the management of women with rare monogenic diseases during pregnancy, delivery, and the postpartum period is scarce.^1^ The lack of knowledge and evidence‐based recommendations about this topic, however, put women and/or their offspring at a substantial health risk. This is demonstrated by potentially life‐threatening hyperammonemic encephalopathy in untreated women with ornithine transcarbamylase deficiency^2^ and the manifestation of maternal phenylketonuria syndrome in the offspring of inadequately treated women with phenylketonuria.^3^ Neuroblastoma amplified sequence (NBAS)‐associated disease is a rare condition with yet unknown health risks for pregnant women and their offspring.

NBAS‐associated disease is an autosomal‐recessive condition with a wide and heterogeneous phenotypic spectrum. In 2010, it was first described that a certain homozygous variant in the *NBAS* gene leads to a clinical syndrome with short stature, optic atrophy and Pelger‐Huët anomaly (SOPH syndrome; MIM: 614800). In 2015, biallelic pathogenic *NBAS* variants were found in several patients with recurrent acute liver failure (ALF) triggered by febrile infections. By now, there is a total number of over 130 published cases of NBAS‐associated disease worldwide.^4^, ^5^, ^6^, ^7^, ^8^

There are three clinical subgroups of NBAS‐associated disease that are related to the affected protein domains^4^: patients with missense variants affecting the Sec 39 domain present with a predominantly hepatic phenotype, characterized by recurrent ALF triggered by febrile infections, named infantile liver failure syndrome type 2 (ILFS2; MIM: 616483). Patients with missense variants affecting the C‐terminal protein segment show a multisystemic phenotype involving growth, skeleton, integument, immune system, nervous system, endocrine system, eye, and liver. A severe combined phenotype with multisystemic involvement and recurrent ALF is observed in most patients with missense variants affecting the ß‐propeller domain.

Although ALF in NBAS‐associated disease usually manifests in infancy and becomes less frequent with age, it is not restricted to childhood, and there is a remaining risk of decompensation in adulthood. It is still a matter of debate, whether temperature itself or febrile infections trigger decompensation in NBAS‐associated disease; however in vitro data suggest that elevated temperature leads to a further decrease of NBAS protein expression level, increases ER stress and thereby potentially triggers decompensation.^9^, ^10^

## 2 METHODS

Patient recruitment was coordinated through the Division of Pediatric Neurology and Metabolic Medicine, Center for Child and Adolescent Medicine of the University Hospital Heidelberg and the Center of Obstetrics and Gynecology at the University of Marburg. All procedures were in accordance with the ethical standards of the responsible committee on human experimentation and with the Helsinki Declaration of 1975, as revised in 2013. Informed consent to participate in the study was obtained from the patient. The study was approved by the ethical committee of the University Hospital Heidelberg (S‐035/2014).

Parameters included maternal medical history (genotype, hepatic phenotype, extrahepatic symptoms), maternal clinical and laboratory parameters during pregnancy, delivery and postpartum, mode of birth, disease‐specific monitoring or therapy, and fetal data (growth during pregnancy, birth data).

## 3 RESULTS

**FIGURE 1:**
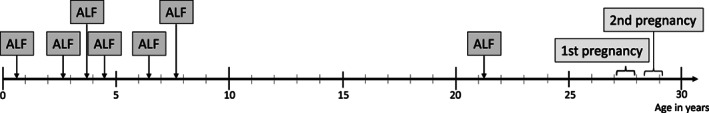
Timeline of acute liver failure (ALF) episodes and pregnancies: Although ALF mainly manifested in infancy, the patient suffered her last ALF episode at 21 years of age, 6 years prior to her first pregnancy.

We followed two pregnancies of a 30‐year‐old woman with recurrent ALF due to the homozygous missense *NBAS* variant c.2708 T > G (p.Leu903Arg) affecting the Sec39 domain of NBAS. She was among the first patients in whom biallelic *NBAS* variants were identified as a cause of recurrent ALF.^11^ Revising her medical history, she experienced seven episodes of ALF and at least nine further episodes with elevated alanine and aspartate aminotransferases (ALAT, ASAT), all of them precipitated by febrile infections. The first ALF occurred at the age of 7 months, her last ALF aged 21 years (Figure 1). During her last ALF, the patient developed acute renal failure requiring hemodialysis. Just as liver function always recovered between crises, kidney function showed complete normalization. A normal liver ultrasound and more specifically normal results in liver transient elastography during the first pregnancy (gestational age 23 + 3 weeks) indicate that the recurrent liver crises did not result in hepatic fibrosis. Extrahepatic symptoms in this patient included epileptic seizures until the age of 12 years (treated with Phenobarbital and Sultiam, antiepileptic therapy ended at the age of 15 years), short stature (height 157 cm, −1.8 SD; parental height 0.69 SD), mild facial anomalies (hypotelorism, sloping lid axis, flat cheekbones), mild brain atrophy (MRI) and reduced NK cell number and function. Altogether, the patient shows a phenotype characteristic for ILFS2.

During both pregnancies (at the ages of 27 years and 28 years), the mother was managed by the perinatal team of the University Hospital Marburg in close collaboration with disease experts from the Division of Pediatric Neurology and Metabolic Medicine, Center for Child and Adolescent of the University Hospital Heidelberg.

### 3.1 Pregnancies

Time to pregnancy (defined as time to proof of pregnancy after start of regular sexual intercourse without contraception) was below 6 months in both pregnancies, there was no fertility treatment and no history of miscarriage. The interval between first birth and second pregnancy was 10 months and both pregnancies were planned. Genetic counseling was provided for the parents. Given the low prevalence of NBAS‐associated disease and the fact that there is no parental consanguinity, the parents decided against a testing for paternal carriership. Except for vaginal candidiasis at 13 weeks of gestation during the first pregnancy and minor vaginal bleeding at 17 + 4 weeks of gestation during the second pregnancy, there were no complications related to pregnancy. Febrile infections did not occur during both pregnancies and regular blood tests every 4 weeks showed normal hepatic transaminases as well as normal liver function. Fetal development was monitored by regular ultrasonographic follow‐up showing regular fetal growth between the 25th and 90th percentile and no morphological abnormalities in both pregnancies.

### 3.2 Childbirth

At 40 + 4 weeks of gestation during the first pregnancy, the cardiotocography (CTG) monitoring showed a suspicious CTG with a variable deceleration. To avoid prolonged vaginal delivery, medical induction with a Cook catheter followed by oxytocin and as an alternative a cesarean section were discussed with the patient. Decision was in favor of the cesarean section, which was performed without complications. Spinal anesthesia was administered with bupivacaine at standard doses and a single shot of cefuroxime was applied. A boy was born (3550 g (−0.31 SD), supine length 53 cm (0.07 SD), head circumference 34 cm (−1.36 SD)), and postpartum adaption was without complications (APGAR 9/10/10, pH 7.34).

For the second childbirth, the mother decided for a primary cesarean section, which was performed at 38 + 3 weeks of gestation. Perioperative management was identical to the first cesarean delivery. Another boy was born showing normal postnatal adaptation to extra‐uterine life (3190 g (−0.45 SD), supine length 50 cm (−0.63 SD), head circumference 35 cm (−0.06 SD), APGAR 9/10/10, pH 7.30).

### 3.3 Postpartum period

**FIGURE 2:**
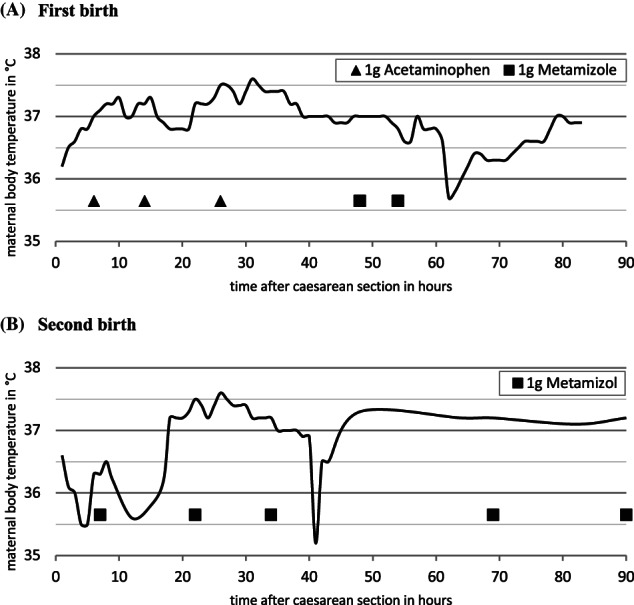
Maternal body temperature after caesarian section: Under antipyretic, respectively analgesic treatment, maternal body temperature remained below 38°C.

Following both deliveries the woman was monitored at the obstetric intermediate care unit, with hourly determination of body temperature for 36 hours (Figure 2) and regular blood samples to monitor liver function (Table 1). Increases of body temperature above 37.5°C were immediately managed with acetaminophen and metamizole. There was no elevation of ALAT or ASAT. Considering the increased risk of fever during lactation, especially early lactation, the mother was advised to ablactate, which was done using 1 g of cabergolin on the first day after delivery. Mother and child were transferred to the obstetric ward after 2–3 days after delivery and were discharged after 4 days without complications.

**TABLE 1:** Laboratory monitoring before and after caesarian section

First delivery										
	Preoperative	Hours after Caesarian section							Reference range	Unit
		7 h	13 h	22 h	30 h	39 h	45 h	53 h		
ASAT	21	24	27	29	25	24	21	22	<35	U/l
ALAT	12	9	7	9	10	8	7	9	<35	U/l
INR	0.84		0.85	**0.84**		**0.83**			0.85–1.15	ratio
Glucose	73	79	**133**	79	**150**	**122**	**113**	95	65–110	mg/dl
LDH	174	**306**	**340**	**354**	239	241	239	218	<248	U/l
CRP	1.7	4.1	**13.2**	**40.8**	**71.1**	**82.8**	**68.1**	**58.1**	<5	mg/l
WBC	9.3	**12.4**	**12.5**	**10.3**	**11.0**	**14.2**	**11.9**	**11.2**	4–10	/nl

Second delivery										
		10 h	18 h	72 h						
ASAT	19	20	22	18					<35	U/l
ALAT	12	10	10	8					<35	U/l
INR	0.85								0.85–1.15	ratio
Glucose	91	**135**	85	94					65–110	mg/dl
LDH	167	212	230						<248	U/l
CRP	1.8	**17.5**	**54.6**	**62.9**					<5	mg/l
WBC	7.9	**14.3**	**13.9**	**8.9**					4–10	/nl

*Note*: Results outside the reference range in bold type.

Abbreviations: ASAT, aspartate aminotransferase; ALAT, alanine aminotransferase; CRP, C‐reactive protein; h, hours; INR, international normalized ratio; LDH, lactate dehydrogenase; WBC, white blood cells.

### 3.4 Strategy for the management of women with NBAS‐associated diseases during pregnancy, childbirth, and the and postpartum period

Based on this experience, current understanding of NBAS‐associated disease and general obstetrical knowledge, we developed the following recommendations:

- Management in a multidisciplinary team.
- Avoidance or reduction of potential risk factors for fever and infection.
- Close monitoring of body temperature and liver function during pregnancy and especially birth and postpartum.
- Immediate antipyresis if body temperature exceeds 37.5°C.
- In case of risk for decompensation administration of glucose at anabolic dosage.

## 4 DISCUSSION

This is the first report on successful pregnancy and delivery in a woman with NBAS‐associated disease. Avoidance of potential risk factor, collaborative development of a management strategy and careful medical assistance and monitoring by an interdisciplinary team of gynecologists, anesthesiologists, and metabolic experts helped to prevent potentially life‐threatening ALF.

Since fever and febrile infections are the only known triggers of ALF in ILFS2,^4^, ^9^ clinical management aims to prevent fever and infectious disease. Known risk factors for fever during labor that we attempted to eliminate or at least minimize, include epidural analgesia, long duration of labor, and a long interval from the rupture of membranes to delivery.^12^, ^13^, ^14^ Postpartum fever is mainly associated with uterine infections, wound infections, urinary tract infections and breastfeeding. Remarkably, the incidence of postpartum infection is up to five times higher after cesarean delivery than after vaginal delivery, mainly attributed to wound infections and urinary tract infections.^15^ This underlines the importance of careful monitoring for possible infections after cesarean delivery and supports that vaginal delivery may be a reasonable option. In our patient, during postpartum period, an increase of inflammatory markers has been observed. Considering the patients good general condition without fever and without a focus of infection, this was not considered to be a sign of infection, but a physiological stress response after surgical delivery. Apparently, this stress response was not a trigger for a liver crisis in our patient. In general, in patients with ILFS2, frequency and severity of liver crises decrease with age,^9^ indicating a lower risk of liver crises in women of reproductive age compared to childhood.

So far, there has been no systematic evaluation of therapeutic strategies for acute liver failure in NBAS‐associated disease. However, there are reports on early and aggressive antipyretic treatment preventing severe liver crises.^9^, ^16^, ^17^ Receiving acetaminophen and metamizole for antipyretic as well as analgetic purpose, our patient showed normal liver function. Nevertheless, it is unknown, whether there would have been liver dysfunction if fever had been tolerated. The preferred antipyretic medication during the third trimester and peripartum is acetaminophen. Although acetaminophen overdose may itself be a trigger of ALF, it can, at recommended doses, be used safely in patients with liver disease.^18^, ^19^ If the mother is not breastfeeding, metamizole is also a reasonable option for analgesia and antipyresis. In addition, physical methods of reducing body temperature can be considered.

There are reports on parenteral glucose and lipids reducing duration and severity of liver crises.^9^ Our patient received glucose only as a standard infusion since body temperature was controlled under 38 °C and there was no sign of infection. However, continuous prophylactic anabolic glucose administration can be considered during delivery, as there is an increased risk for decompensation. In case of signs of decompensation (fever, infection, elevated transaminases), we strongly recommend anabolic glucose administration (3 g/kg body weight/day).

Pathogenic *NBAS* variants are associated with different phenotypes. Therefore, observations and findings from this patient cannot be generalized for the C‐terminal and ß‐propeller subgroups, both presenting with a primarily multisystemic involvement. Endocrinological abnormalities, skeletal dysplasia or immunodeficiency present further concerns for pregnancy and childbirth in these women.

In summary, we present the first study on pregnancies and births in a mother with ILFS2. A short time to pregnancy and normal intrauterine growth indicate a normal function of the female reproductive system. We provide recommendations for disease specific management of labor, childbirth and postpartum period in ILFS2. We cannot conclude that successful pregnancy and birth are not possible without these precautions, however, in our opinion, the potential risks clearly outweigh the associated restrictions.

## FUNDING STATEMENT

The authors declare that no funds, grants, or other support were received related to the study.

## CONFLICT OF INTEREST STATEMENT

Bianca Peters, Felix Wiemers, Dominic Lenz, Stefan Kölker, Georg F. Hoffmann, Siegmund Köhler and Christian Staufner declare that they have no conflict of interest.

## ETHICS STATEMENT

All procedures followed were in accordance with the ethical standards of the responsible committee on human experimentation (institutional and national) and with the Helsinki Declaration of 1975, as revised in 2013 (7). Informed consent was obtained from the patient for being included in the study.  

## Data Availability

The data that support the findings of this study are available from the corresponding author upon reasonable request.
